# Survey on brachytherapy training among radiation oncology residents in the German-speaking regions of Europe

**DOI:** 10.1007/s00066-023-02108-3

**Published:** 2023-07-18

**Authors:** Johannes Knoth, Stefan Konrad, Kristina Lössl, Laura Motisi, Matthias Mäurer, Philipp Linde, Katja Lindel, Peter Niehoff, Vratislav Strnad, Alina Sturdza, Stefanie Corradini

**Affiliations:** 1 Department of Radiation Oncology, Comprehensive Cancer Center, Medical University of Vienna, Währinger Gürel 8–20, 1090 Vienna, Austria; 2 Department of Radiation Oncology, Inselspital, Bern University Hospital, University of Bern, Bern, Switzerland; 3 Department of Radiation Oncology, University Hospital Zurich, University of Zurich, Zurich, Switzerland; 4 Department for Radiotherapy and Radiation Oncology, University Hospital Jena, Friedrich-Schiller-University, Jena, Germany; 5 Clinician Scientist Program “OrganAge”, Jena University Hospital, 07747 Jena, Germany; 6 Department of Radiation Oncology, Cyberknife and Radiation Therapy, Faculty of Medicine and University Hospital of Cologne, University of Cologne, Kerpener St 62, 50937 Cologne, Germany; 7 Department of Radiation Oncology, Klinikum Karlsruhe, Karlsruhe, Germany; 8 Sana Klinikum Offenbach, Offenbach, Germany; 9 Department of Radiation Oncology, University Hospital Erlangen, Erlangen, Germany; 10 Department of Radiation Oncology, University Hospital, LMU Munich, Munich, Germany

**Keywords:** Teaching, Education, Survey, Questionnaire, Career choice

## Abstract

**Purpose:**

This survey aimed to determine the perception of brachytherapy training among residents in the DACH region, consisting of Austria, Germany and Switzerland.

**Material & Methods:**

An online questionnaire containing 22 questions related to trainee demographics (*n* = 5) and to brachytherapy training (*n* = 17) was sent in two iterations in 11/2019 and 02/2020. The following topics were evaluated: institutional support, barriers to training, extent of training, site-specific training (prostate, gynaecology, breast, gastrointestinal and skin), preferences for further training and outlook on overall development of brachytherapy. The responses were mostly based on a Likert scale of 1 to 5, thereby reflecting strength of opinion. Descriptive statistics were used to describe frequencies.

**Results:**

Among the 108 respondents, approximately 69% of residents considered the ability to perform brachytherapy independently to be important or somewhat important. However, only 31% of respondents reported to have a dedicated brachytherapy training during residency. The major limitation to achieve independence in performing brachytherapy was seen in a low case load in Austria, in the lack of training in Switzerland and in both of them in Germany.

**Conclusion:**

The interest in brachytherapy training among residents in German-speaking countries was generally high, but there is a perceived lack of sufficient case volumes and partially also in formal training opportunities. Fellowships at departments with a high case load as part of a formalised curriculum and dedicated hands-on workshops at national or international conferences might help to overcome these issues.

**Supplementary Information:**

The online version of this article (10.1007/s00066-023-02108-3) contains supplementary material, which is available to authorized users.

## Introduction

Brachytherapy provides essential benefits in the treatment of various tumors such as locally advanced cervical cancer [1], prostate cancer [2], adjuvant or recurrent breast cancer [3], and others. As a subspecialty of radiotherapy, it is not available at every radiation oncology center and is thus probably often taught only partially or not at all. The looming lack of training is potentially aggravated by less frequent use of brachytherapy in some parts of the world [4–7].

Four surveys have recently been performed among radiation oncology residents to evaluate the state of brachytherapy training in the US [8], in Australia/New Zealand [9], and in Europe [10, 11]. Senior residents were confident to join a brachytherapy practice in 54% (US) and 34% (Europe). The greatest barrier to achieving independence in performing brachytherapy was a low case load in the US as well as in Australia/New Zealand, and the lack of appropriate didactic/procedural training in Europe.

Radiation oncology training differs between European countries and until recently, little was known about the perception and needs of trainees. The aim of this study was to provide a more differentiated view of the current state of brachytherapy training and on its perception by trainees in the DACH region of Germany (D = GER), Austria (A = AUT), and Switzerland (CH = SUI). In this way, possible opportunities for improvement could be identified.

## Materials and methods

An online questionnaire was developed to evaluate the brachytherapy training situation in Europe. It was based on the survey conducted by the American Association of Radiation Oncology Residents (ARRO) in 2017 with the addition of questions unique to training in Europe, and contained 22 questions related to trainee demographics (*n* = 5) and to training itself (*n* = 17) [8]. A positive vote from the ethical review committees in Vienna and Munich was deemed unnecessary for questionnaires in general. The following topics were evaluated: institutional support, barriers to training, extent of training, site-specific training (prostate, gynecology, breast, gastrointestinal, and skin), preferences for further training, and outlook on development of brachytherapy. A detailed overview of the questions can be seen online (https://wumarketing.eu.qualtrics.com/jfe/form/SV_2g8RFFlYJpqdsNL) or in the supplementary information.. Most questions were based on a Likert scale of 1 to 5, thereby reflecting strength of opinion. The questionnaire was sent out in two iterations: in November 2019 (personally within a meeting of the young Austrian Society of Radiation Oncology [youngÖGRO]) and in February 2020 online for all residents from AUT, GER, and SUI. Answers were categorized by country (AUT, GER, SUI) as well as by level of training (junior summarizing residents with ≤ 3 years of training vs. senior summarizing residents with 4–6 years of training and junior staff following their residency training). Descriptive statistics were used to describe frequencies.

## Results

### Participants

The survey was answered by 108 of 338 invited radiation oncology trainees from Austria (*n* = 39/74), Germany (*n* = 58/180), and Switzerland (*n* = 11/84), which equals an overall response rate of 32% (AUT: 53%, GER: 32%, SUI: 13%). The median age of participants was 32 years (range 25–50 years), with 28 females, 44 males, and 36 of unknown gender (AUT: 1 female, 2 males, 36 unknowns; GER: 18 females, 40 males; SUI: 9 females, 2 males). The level of radiation oncology training for the overall population consisted of 41 junior and 67 senior participants (AUT: 16 juniors and 23 seniors; GER: 19 juniors and 39 seniors; SUI: 6 juniors and 5 seniors).

### General aspects

The ability to perform brachytherapy independently was considered important or somewhat important by about 69% of the residents, with decreasing percentage from junior to senior level (Table 1). However, a formal brachytherapy curriculum was reported by only about 31% of the trainees. Overall, 47% of the respondents felt that performing brachytherapy independently was valued by their residency program. This feeling was most prominent in AUT (64%) and least prominent in SUI (27%), and decreased from junior to senior level. Nevertheless, only 22% of residents report a formal brachytherapy training evaluation.

**Table 1:** Comparison between selected responses of residents: overall and by junior and senior training year(s) as well as by country

Responses of the surveyed residents	Percentage of trainees, overall (%)	Percentage of junior trainees (%)	Percentage of senior trainees (%)	Percentage of Austrian trainees (%)	Percentage of German trainees (%)	Percentage of Swiss trainees (%)
% of residents who think that performing BT independently at the end of residency is “very or somewhat” important	69	76	66	85	62	55
% of residents who think that decreasing utilization of BT is “very troubling” or “troubling”	40	46	36	46	35	46
% of residents who think that performing BT independently is valued by their residency program	47	56	42	64	40	27
% who think a 15 cases intracavitary requirement is sufficient to gain confidence in gynecological BT	45	37	51	49	41	55
% who that think a 5 cases interstitial requirement is sufficient to gain confidence in gynecological BT	12	2	15	18	7	0
% who strongly agree or agree to having a formal BT curriculum	31	29	31	33	28	36
% of residents who have formal BT training evaluation	22	15	27	21	22	27
Greatest barrier to achieving BT independence at the end of residency—lack of training	31	37	28	21	36	45
Greatest barrier to achieving BT independence at the end of residency—low volume	35	32	37	46	31	18
Greatest barrier to achieving BT independence at the end of residency—lack of interest	10	10	10	0	17	9
High or somewhat high confidence to start a BT practice at the end of residency	19	15	22	33	12	9
High or somewhat high confidence to start a SBRT practice	45	44	46	44	43	64
Total respondents	108	41	67	39	58	11

*BT* Brachytherapy, *SBRT* Stereotactic body radiotherapy

### In-depth features

**Fig. 1:**
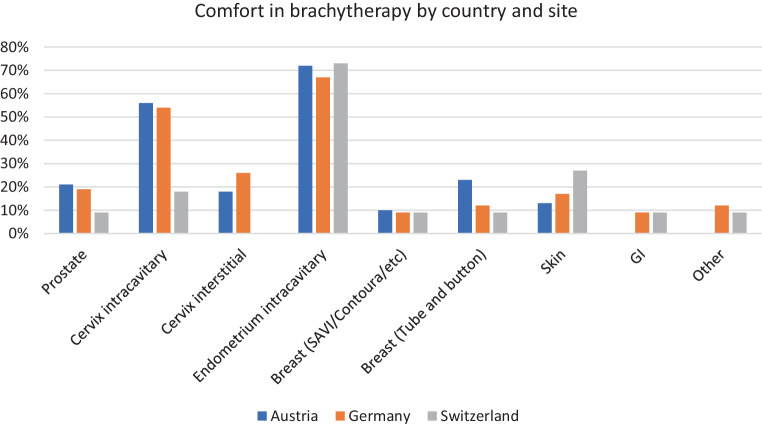
Percentage of respondents feeling highly likely or likely to be confident in performing brachytherapy (BT) at the end of residency based on the respective BT sites for residents in Austria (*blue*), Germany (*orange*), and Switzerland (*grey*). (Sites “GI” and “Other” were not part of the questionnaire in Austria). *GI* Gastrointestinal, *SAVI* ”Strut Adjusted Volume Implant“ (Merit Medical, USA), Contoura SenoRx, USA

**Fig. 2:**
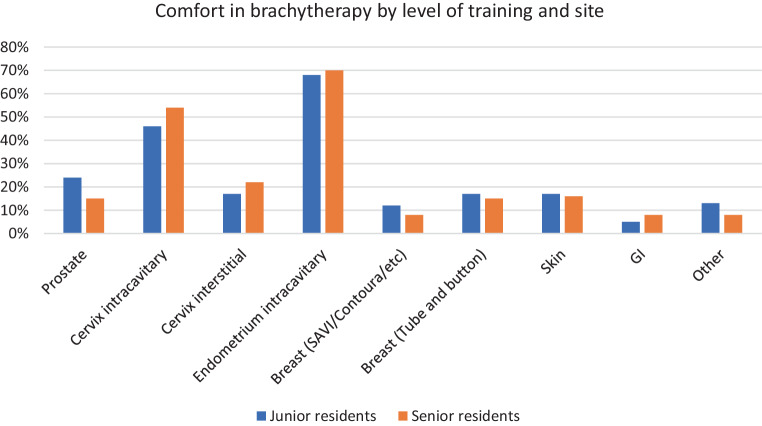
Percentage of respondents feeling highly likely or likely to be confident in performing brachytherapy (BT) at the end of residency based on the respective BT sites for junior residents (*blue*) and senior residents (*orange*). *GI* Gastrointestinal, *SAVI* ”Strut Adjusted Volume Implant“ (Merit Medical, USA), Contoura SenoRx, USA

Regarding the clinical experience required, only 12% of respondents believe that five cases of interstitial applications are sufficient to gain confidence in gynecological brachytherapy. The experience of performed brachytherapy cases per site was generally low (highest: vaginal cylinder applications for postoperative endometrial cancer; lowest: surface applications for skin cancer) and increased from junior to senior level only in 1) vaginal cylinder applications for postoperative endometrial cancer and in a smaller magnitude in 2) intracavitary applications for cervical cancer and 3) combined intracavitary/interstitial applications for cervical cancer (Table 2). According to these findings, respondents felt highly likely or likely confident in performing brachytherapy only in vaginal cylinder applications for postoperative endometrial cancer and partially in intracavitary applications for cervical cancer (at least in AUT and GER; Fig. 1). In contrast, in prostate, breast, and skin brachytherapy, the confidence actually decreased from junior to senior level (Fig. 2). The general confidence to start a brachytherapy practice after residency was high or somewhat high in only about 19% of residents, with a slightly higher confidence in AUT compared to GER and SUI (Table 1). The greatest barrier to achieving independence in performing brachytherapy was seen in a low caseload in AUT, in lack of training in SUI, and in both in GER.

**Table 2:** Current number of BT cases performed during training by site and applicator type as well as by level of training and by country

Question	Juniors, % (*N* = 34)		Seniors,% (*N* = 58)		Austrians,% (*N* = 32)		Germans, % (*N* = 52)		Swiss, % (*N* = 8)	
Current number of cases you have performed in your training	≤ 5	> 5	≤ 5	> 5	≤ 5	> 5	≤ 5	> 5	≤ 5	> 5
Definitive prostate (LDR or HDR)	82	18	86	14	81	19	85	15	100	0
Definitive cervix intracavitary	82	18	64	36	69	31	69	31	88	12
Definitive cervix combined intracavitary/interstitial	88	12	72	28	76	24	79	21	88	12
Postoperative endometrial vaginal cylinder	65	35	38	62	41	59	54	46	38	62
Adjuvant breast treatment after lumpectomy (SAVI^a^, Contoura^b^, Mammosite^c^)	94	6	98	2	91	9	100	0	100	0
Adjuvant breast treatment after lumpectomy (interstitial tube and button)	91	9	88	12	78	12	96	4	88	12
Nonmelanoma skin cancer (applicator like Valencia/Leipzig/Xoft^d^/Esteya^e^)	100	0	100	0	100	0	100	0	100	0
Gastrointestinal (esophageal/rectal cancer)	100	0	88	12	80	20	92	8	100	0
Other, specify^f^	96	4	88	12	100	0	90	10	88	12

*LDR* low dose rate, *HDR* high dose rate

^a^*SAVI* “Strut Adjusted Volume Implant” (Merit Medical, USA)

^b^SenoRx, USA

^c^Hologic, USA

^d^iCAD, USA

^e^Elekta AB, Sweden

^f^“others” were specified by participants as liver and eye

### Training and future role of brachytherapy

The modality perceived most important for improved training was a skills lab (55% of respondents) followed by online teaching modules (20%), national society teaching courses (13%), more continuing educational sessions (7%), and others (5%). The mentioned training modalities were “highly likely” or “likely” to be pursued by residents during the residency training, ESTRO school courses, or national brachytherapy courses (Table 3).

**Table 3:** Percentage of respondents “highly likely” or “likely” to pursue different teaching options displayed overall and by country

If you did not achieve independence during residency in brachytherapy, how likely would you be to pursue the following options?				
	Overall (in %)	Austria (in %)	Germany (in %)	Switzerland (in %)
GEC ESTRO workshop	44	53	39	38
ESTRO school	62	71	55	63
Fellowship	35	44	27	50
On-job training	70	85	59	75
National BT course	52	29	53	63
Other	5	0	6	0

*BT* Brachytherapy

**Fig. 3:**
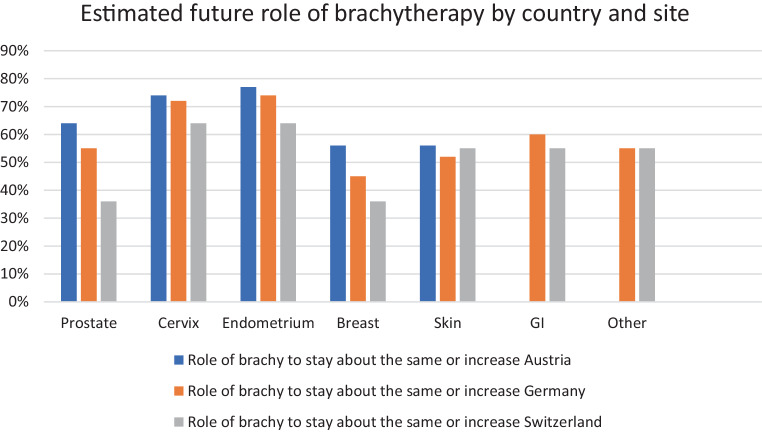
Estimation of the future role of brachytherapy for residents in Austria (*blue*), Germany (*orange*), and Switzerland (*grey*). (Sites “GI” and “Other” were part of the questionnaire in Austria). *GI* Gastrointestinal

The future role of brachytherapy was expected to stay about the same or to increase by more than 50% of respondents regarding endometrial, cervical, prostate (except for SUI), skin, and gastrointestinal (GI) brachytherapy (Fig. 3), whereas the decreasing utilization of brachytherapy was considered “troubling” or “somewhat troubling” by 40% of residents.

## Discussion

To our knowledge, this is the first survey summarizing the perception of brachytherapy training and its limitations in German-speaking countries (DACH). The importance of independently performing brachytherapy was reported by the majority of trainees. However, only one third of all respondents reported having a formal brachytherapy curriculum and only one fifth a formal evaluation of their brachytherapy training during residency. The requirements for the radiation oncology specialist examination (*Facharzt*) in AUT, where residents have to see at least 20 brachytherapy treatments within 6 years of training, and in GER, where residents must have performed at least 100 brachytherapy treatments, seem to be met (otherwise, no candidates could register for the examination). The required number of intracavitary implants in SUI (*n* = 5) is comparably low. Nevertheless, based on this survey, it must be assumed that the cases seen consist predominantly of “simple” intracavitary interventions.

The greatest barriers to achieving independence were seen in a low case volume (AUT, GER) as well as in a lack of formal training (GER, SUI), which resulted in low confidence to start a brachytherapy practice (only 22% of senior trainees). These findings are similar to recently published results based on data of 437 respondents from a European survey [10]. In Europe, the lack of formal training (49% of responders) and a low caseload (31% of responders) were seen as the main limitations in brachytherapy training. The confidence in starting a brachytherapy practice in Europe was only slightly higher compared to German-speaking countries (34% vs. 22% of seniors) but still lower compared to the USA, where 54% of residents felt comfortable to start a practice, although an adequate case volume was considered a problem [8]. Similarly, residents in Australia and New Zealand found case volume to be the greatest barrier in brachytherapy training, but about 60% of them considered themselves capable of performing brachytherapy as part of their future clinical practice [9].

Regarding disease-specific sites, only intracavitary applications for postoperative endometrial cancer were performed more than five times by senior residents in our cohort. This is again in line with the European findings [10] and the findings from residents in the US and Australia/New Zealand [8, 9]. At the same time, only 12% of respondents in our cohort believed that five cases of combined intracavitary/interstitial applications for cervical cancer were sufficient to gain confidence in gynecological brachytherapy. This discrepancy between demanded and actually performed cases results in low levels of confidence. Accordingly, only intracavitary applications for endometrial and cervical cancer were seen as “likely” or “highly likely” to be comfortably performed by more than 50% of trainees in our cohort. Other disease sites such as prostate, breast, GI, skin, and generally all interstitial applications were outside the comfort zone of the senior residents. Similarly, all of the aforementioned sites were seen as “likely” or “highly likely” to be comfortably performed by less than 50% of trainees from Australia/New Zealand [9]. The self-confidence of residents from the US was generally higher, so that almost all participants felt able to perform intracavitary procedures comfortably, and also prostate implants as well as combined intracavitary/interstitial cervical procedures were not seen as a difficulty by the majority. However, this is in contrast to the perceived low caseload [8].

Different approaches have been undertaken to overcome the abovementioned issues. For example, the required number of tandem-based applications in the US has been elevated [8, 12–14]. Furthermore, residents in the US are given the opportunity to take part in gynae fellowships within the “300-in-10” initiative, with the goal of training 300 brachytherapists in the next 10 years [13–17]. Similarly, Canadian radiation oncologists can enroll in an accredited 12-month brachytherapy program at certified brachytherapy centers after passing their fellowship examination [18]. Another approach has been to offer simulation-based workshops, especially for gynecologic and prostate brachytherapy [14, 15, 19–26]. In Europe, the European Society for Radiotherapy and Oncology (ESTRO) offers teaching courses and the Groupe Européen de Curiethérapie (GEC)-ESTRO as well as the EMBRACE study group have started educational initiatives [13–15, 23, 27–36]. Similar initiatives are offered in GER. Since 2009, biannual or annual “Basics of Brachytherapy” courses have been organized by members of the Brachytherapy Working Group of the German Society of Radiation Oncology (DEGRO). To date, 22 courses have taken place. These courses offer back-to-back a special course on brachytherapy of a specific disease site (e.g., breast, gynae, prostate, liver, and others). Moreover, in 2022, the DEGRO brachytherapy working group held the first hands-on brachytherapy workshop for members of the youngDEGRO during the annual DEGRO conference. In addition, every other year, a BT congress of the three German-speaking countries takes place with dedicated time for updates on BT, with high participation of young trainees.

This is consistent with the preferred teaching modalities for the majority of respondents in our cohort: a skills lab, followed by online teaching courses and national society teaching courses, while they would most likely improve their education in on-the-job-training, ESTRO courses, or national society teaching courses. To fulfil these needs, a centralization of brachytherapy patients as already partly done in SUI could help to provide a sufficient case volume within on-the-job-training. Simultaneously, fellowships similar to those in the USA or Canada might be beneficial to connect fellows and institutions with high caseloads. Residents in SUI are, for example, obliged to rotate to another department for one year and are thereby able to choose one with a high case volume in brachytherapy, while Spanish residents have to rotate to a brachytherapy department. Uni- or bilateral rotations between departments with different (brachytherapy) foci in the DACH region might be considered similarly. The DEGRO brachytherapy working group has recently launched an observership/internship program together with the youngDEGRO (https://www.degro.org/jd/brachytherapie-programm/). It offers the opportunity to spend time at facilities with high caseloads of brachytherapy patients and gain practical experience. Additionally, dedicated simulation-based workshops might be established at national and European conferences.

There are some limitations to our survey apart from the questionnaire-inherent recall bias. The response rate differed between countries (53% in AUT, 32% in GER, 13% in SUI), and might in part lead to a selection bias of respondents. Respondents themselves could furthermore either over- or underestimate themselves during self-assessment. Finally, single procedural steps such as applicator selection or target delineation have not been considered within this survey.

## Conclusion

The interest in brachytherapy training among residents in German-speaking countries was generally high, but there is a perceived lack of case volumes and partially in formal training opportunities. Fellowships at departments with a high caseload as part of a formalized curriculum and dedicated hands-on workshops at conferences might help to overcome these issues. There are already some ongoing initiatives at the national and international level, but these need to be expanded to inspire future brachytherapists and to provide structured training for our next generation.

## Supplementary Information

The questionaire which was distributed for this evaluation can be found here.

## Abbreviations

ARRO: American Association of Radiation Oncology Residents
AUT: Austria
DACH: Germany, Austria, Switzerland
DEGRO: German Society of Radiation Oncology
ESTRO: European Society for Radiotherapy and Oncology
GEC-ESTRO: Groupe Européen de Curiethérapie of the ESTRO
GER: Germany
GI: Gastrointestinal
SUI: Switzerland
US: United States of America
youngDEGRO: Young members of the German Society of Radiation Oncology
youngÖGRO: Young members of the Austrian Society of Radiation Oncology
