# Supplementary material for: Survey on brachytherapy training among radiation oncology residents in the German-speaking regions of Europe
Source: Strahlenther Onkol. 2023 Jul 18;199(9):798–805. doi: 10.1007/s00066-023-02108-3 (PMC10450005; doi:10.1007/s00066-023-02108-3)
Supplement: Supplementary file 1 — The questionaire which was distributed for this evaluation can be found here. [file 66_2023_2108_MOESM1_ESM.docx]

| **Total number of Responders** |  |  |  | | | | | |
| --- | --- | --- | --- | --- | --- | --- | --- | --- |
| **Current Year of Training** | **Year 1** | **Year 2** | **Year 3** | **Year 4** | **Year 5** | **Year 6** | Junior specialist |  |
|  |  |  |  |  |  |  |  |  |
| **Which specialty are you training in?** |  | **Radiation Oncology** | **Clinical Oncology** |  | | |  |  |
|  |  |  |  |  |  |  |  |  |
| Country of training |  |  | | | | | | |
| Gender |  | Female: Male: NA: |  |  |  |  |  |  |
| Age |  |  |  |  |  |  |  |  |
| **I feel that being able to independently perform brachytherapy by the end of my residency is:** |  | **Very important** | **Somewhat important** | **Neutral** | **Somewhat unimportant** | **Not important** |  |  |
|  |  |  |  |  |  |  |  |  |
| **I feel that my residency program director and chair place value on my being able to perform brachytherapy independently by the end of my residency.** |  | **Strongly agree** | **Agree** | **Neutral** | **Disagree** | **Strongly disagree** |  |  |
|  |  |  |  |  |  |  |  |  |
|  |  |  |  |  |  |  |  |  |
| **The chances of you comfortably performing brachytherapy independently for the following disease sites by the end of your residency is:** |  | **Highly likely** | **Likely** | **Neutral** | **Unlikely** | **Highly unlikely** |  |  |
| Definitive Prostate (LDR or HDR) |  |  |  |  |  |  |  |  |
| Post-operative Endometrial Vaginal cylinder |  |  |  |  |  |  |  |  |
| Adjuvant breast treatment after lumpectomy (SAVI, Contura, Mammosite) |  |  |  |  |  |  |  |  |
| Adjuvant breast treatment after lumpectomy (Interstitial tube and button) |  |  |  |  |  |  |  |  |
| Non Melanoma Skin Cancer (applicator like Valencia/Leipzig/Xoft/Esteya) |  |  |  |  |  |  |  |  |

| **Current Number of Cases you have performed in your training:** | NA | 0 | **1-5** | **6-10** | **11-20** | **21-30** | **>30** |
| --- | --- | --- | --- | --- | --- | --- | --- |
| Definitive Prostate (LDR or HDR) |  |  |  |  |  |  |  |
| Definitive Cervix Intracavitary |  |  |  |  |  |  |  |
| Definitive Combined Intracavitary/Interstitial |  |  |  |  |  |  |  |
| Post-operative Endometrial Vaginal cylinder |  |  |  |  |  |  |  |
| Adjuvant breast treatment after lumpectomy (SAVI, Contura, Mammosite) |  |  |  |  |  |  |  |
| Adjuvant breast treatment after lumpectomy (Interstitial tube and button) |  |  |  |  |  |  |  |
| Non Melanoma Skin Cancer (applicator like Valencia/Leipzig/Xoft/Esteya) |  |  |  |  |  |  |  |
| Gastrointestinal (esophageal/Rectal cancer) |  |  |  |  |  |  |  |
| Other, Specify |  |  |  |  |  |  |  |
|  | | | | | | | |
| **I feel that after performing more than 5 interstitial implants that I can independently perform interstitial implants:** |  | **Strongly agree** | **Agree** | **Neutral** | **Disagree** | **Strongly disagree** |  |
|  |  |  |  |  |  |  |  |
|  |  |  |  |  |  |  |  |
| **I feel that after performing more than 15 intracavitary implants that I can independently perform intracavitary implants:** |  | **Strongly agree** | **Agree** | **Neutral** | **Disagree** | **Strongly disagree** |  |
|  |  |  |  |  |  |  |  |
|  |  |  |  |  |  |  |  |
| **If you did not achieve independence during residency in brachytherapy, how likely would you be to pursue the following options?** |  | **Highly likely** | **Likely** | **Undecided** | **Unlikely** | **Highly unlikely** |  |
| GEC ESTRO Workshops |  |  |  |  |  |  |  |
| ESTRO School (e.g. courses: Prostate, Gyneacology, Comprehensive Brachytherapy) |  |  |  |  |  |  |  |
| Fellowship |  |  |  |  |  |  |  |
| On the job training with another member of your group |  |  |  |  |  |  |  |
| National Brachytherapy course |  |  |  |  |  |  |  |
| None of the Above |  |  |  |  |  |  |  |
| Other; please specify |  |  |  |  |  |  |  |
|  | | | | | | |  |
| **Residency Program’s Greatest Barrier to Achieving Independence in Brachytherapy** |  | **Case load at my residency program** | **Lack of appropriate didactic/procedural training from attendings** | **Personal lack of interest in brachy independence** |  |  |  |
|  |  |  |  |  |  |  |  |
|  |  |  |  |  |  |  |  |
| **My ability to independently perform brachytherapy has been formally evaluated by my residency program.** |  | **Strongly agree** | **Agree** | **Neutral** | **Disagree** | **Strongly disagree** |  |
|  |  |  |  |  |  |  |  |
|  |  |  |  |  |  |  |  |
| **My residency has a formal process/curriculum to teach brachytherapy.** |  | **Strongly agree** | **Agree** | **Neutral** | **Disagree** | **Strongly disagree** |  |
|  |  |  |  |  |  |  |  |
|  |  |  |  |  |  |  |  |
| **Do you feel that brachytherapy should be its own subspecialty with formalized training?** |  | **Strongly agree** | **Agree** | **Neutral** | **Disagree** | **Strongly disagree** |  |
|  |  |  |  |  |  |  |  |
| **If you joined a practice after residency where you needed to develop a brachytherapy practice your confidence in being able to accomplish this is:** |  | **High** | **Somewhat high** | **Neutral** | **Somewhat low** | **Low** |  |
|  |  |  |  |  |  |  |  |
|  |  |  |  |  |  |  |  |
| **If you joined a practice after residency where you needed to develop a SBRT/SRS practice your confidence in being able to accomplish this is:** |  | **High** | **Somewhat high** | **Neutral** | **Somewhat low** | **Low** |  |
|  |  |  |  |  |  |  |  |
|  |  |  |  |  |  |  |  |
| **For each of the following disease sites please provide your assessment regarding the role of brachytherapy in the next 10 years.** |  | **Likely increase** | **Stay about the same** | **Likely decrease** |  |  |  |
| Definitive Prostate |  |  |  |  |  |  |  |
| Definitive Cervix |  |  |  |  |  |  |  |
| Post-operative Endometrial |  |  |  |  |  |  |  |
| Adjuvant Breast Treatment After Lumpectomy |  |  |  |  |  |  |  |
| Non-Melanoma Skin Cancer |  |  |  |  |  |  |  |
| Gastrointestinal (esophageal/Rectal cancer) |  |  |  |  |  |  |  |
| Other, please specify |  |  |  |  |  |  |  |
|  |  | | | | | | |
| **The declining utilization of brachytherapy for multiple disease sites is:** |  | **Very troubling** | **Somewhat troubling** | **Neutral** | **Not troubling** |  |  |
|  |  |  |  |  |  |  |  |
